# Interleukin 32 as a Potential Marker for Diagnosis of Tuberculous Pleural Effusion

**DOI:** 10.1128/spectrum.02553-21

**Published:** 2022-07-26

**Authors:** Juan Du, Ming-Ming Shao, Feng-Shuang Yi, Zhong-Yin Huang, Xin Qiao, Qing-Yu Chen, Huan-Zhong Shi, Kan Zhai

**Affiliations:** a Department of Respiratory and Critical Care Medicine, Beijing Institute of Respiratory Medicine and Beijing Chao-Yang Hospital, Capital Medical University, Beijing, China; b Clinical Center for Pleural Diseases, Capital Medical University, Beijing, China; University of Cincinnati

**Keywords:** pleural effusion, tuberculosis, interleukin 32, adenosine deaminase, diagnosis

## Abstract

Accurate differential diagnosis is the key to choosing the correct treatment for pleural effusion. The present study aimed to assess whether interleukin 32 (IL-32) could be a new biomarker of tuberculous pleural effusion (TPE) and to explore the biological role of IL-32 in TPE. IL-32 levels were evaluated in the pleural effusions of 131 patients with undetermined pleural effusion from Wuhan and Beijing cohorts using an enzyme-linked immunosorbent assay method. Macrophages from TPE patients were transfected with IL-32-specific small interfering RNA (siRNA), and adenosine deaminase (ADA) expression was determined by real-time PCR and colorimetric methods. With a cutoff value of 247.9 ng/mL, the area under the curve of the receiver operating characteristic (ROC) curve for IL-32 was 0.933 for TPE, and the sensitivity and specificity were 88.4% and 93.4%, respectively. A multivariate logistic regression model with relatively good diagnostic performance was established. IL-32-specific siRNA downregulated ADA expression in macrophages, and IL-32γ treatment significantly induced ADA expression. Our results indicate that IL-32 in pleural effusion may be a novel biomarker for identifying patients with TPE. In addition, our multivariate model is acceptable to rule in or rule out TPE across diverse prevalence settings. Furthermore, IL-32 may modulate ADA expression in the tuberculosis microenvironment. (This study has been registered at ChiCTR under registration number ChiCTR2100051112 [https://www.chictr.org.cn/index.aspx].)

**IMPORTANCE** Tuberculous pleural effusion (TPE) is a common form of extrapulmonary tuberculosis, with manifestations ranging from benign effusion with spontaneous absorption to effusion with pleural thickening, empyema, and even fibrosis, which can lead to a lasting impairment of lung function. Therefore, it is of great significance to find a rapid method to establish early diagnosis and apply antituberculosis therapy in the early stage. This study indicates that interleukin 32 (IL-32) in pleural effusion is a new high-potency marker to distinguish TPE from pleural effusions with other etiologies. A multivariate model combining age, adenosine deaminase (ADA), lactic dehydrogenase, and IL-32 may reliably rule in TPE in intermediate- or high-prevalence areas. Additionally, we observed that IL-32 might regulate ADA expression in macrophages in the tuberculosis microenvironment. Therefore, this study provides new insights into the role of IL-32 in the tuberculosis microenvironment.

## INTRODUCTION

Tuberculous pleural effusion (TPE) is the second most common form of extrapulmonary tuberculosis (1), with presentations ranging from benign effusions that are absorbed spontaneously to complicated effusions with pleural thickening, empyema, and even pleural fibrosis, all of which may result in lasting lung function impairment (2). Early diagnosis and treatment are critical for preventing severe complications of TPE. A definitive diagnosis of TPE requires the isolation and/or culture of Mycobacterium tuberculosis (*Mtb*) from pleural effusions and pleural biopsy specimens or the demonstration of granulomas by pleural biopsy (3). However, due to the low sensitivity of detection of *Mtb* in pleural effusions (10 to 70%) or other specimens (30 to 80%) (2) and the invasive nature of and technical difficulty in medical thoracoscopy, pleural effusion tests have been suggested as an alternative method to diagnose TPE (4). In addition, adenosine deaminase (ADA) can be quantified by a rapid, cost-effective assay and is recognized as a valuable tool in establishing a presumptive diagnosis (5).

Interleukin 32 (IL-32) is a recently discovered proinflammatory cytokine that plays a vital role in the immune response to intracellular pathogens such as *Mtb* (6, 7). IL-32 was identified in natural killer cells and T cells as natural killer cell transcript 4 (8), and more than nine splicing variants have been reported. The major isoforms are IL-32α, IL-32β, and IL-32γ, which have different functions (9). IL-32β is abundant inside cells, while IL-32γ is the most active isoform and the only isoform that can be secreted because the IL-32γ-specific exon contains a signal peptide (10). IL-32 expression was significantly upregulated in the lung tissues of patients with tuberculosis (11) and can induce macrophage apoptosis, a vital killing mechanism of intracellular *Mtb* (12, 13). Those results raise the possibility that the IL-32 level may also have diagnostic value and play an important role in the immune response in TPE.

In this study, the diagnostic efficacy of IL-32 for TPE was evaluated and compared with that of ADA. The immune role of IL-32 in TPE was also further explored. Thus, the current study provides novel insight into the role of IL-32 in the tuberculosis microenvironment.

## RESULTS

### Clinical characteristics.

A total of 27 patients with pleural effusions at Wuhan Union Hospital were enrolled in the discovery study. Subsequently, 104 patients with pleural effusions at Beijing Chao-Yang Hospital were consecutively recruited for the validation study (see Fig. S1 in the supplemental material). Patients with known comorbidities were not included in the discovery study. Apart from comorbidities, there were no significant differences in the demographic characteristics between the two cohorts (Table 1). The age of the patients, the total protein and glucose levels, and the total nucleated cell counts in effusions were significantly different between the TPE and non-TPE (pleural effusions of other etiologies) groups (all *P < *0.01) (Table 2 and Fig. S2). The etiology of each specimen was confirmed by biopsy or thoracoscopic pathology (Table S1). Pathological findings for pleural biopsy specimens from patients with TPE are shown in Table S2.

**TABLE 1:** Demographic characteristics of patients in the studies

Variable	Value for cohort	
	Discovery study (*n* = 27)	Validation study (*n* = 104)
No. of male patients (%)	18 (66.7)	64 (61.5)
Age (yrs)		
Mean ± SEM	52.1 ± 3.9	51.6 ± 2.2
Range	18–86	18–90
No. of patients with known comorbidities (%)^*a*^		38 (36.5)
Diabetes mellitus		8 (7.8)
Hypertension		18 (17.3)
Allergic disease		4 (3.8)
Other cardiovascular problems		11 (10.6)
Other conditions		12 (11.5)

a Several patients have multiple comorbidities.

**TABLE 2:** Clinal, cytological, and biochemical characteristics of pleural effusions in the validation study (*n* = 104)^*a*^

Variable	Value			
	TPE (*n* = 43)	Non-TPE		
		MPE (*n* = 41)	PPE (*n* = 13)	Miscellaneous (*n* = 7)
Mean age (yrs) ± SEM	33.3 ± 2.1***	66.6 ± 2.3	59.5 ± 6.9	61.3 ± 4.8
Mean protein concn (g/L) ± SEM	50.0 ± 1.3**	41.3 ± 1.8	40.2 ± 3.3	26.1 ± 4.8
Mean glucose concn (mmol/L) ± SEM	4.3 ± 0.3**	6.7 ± 0.6	7.2 ± 1.0	7.6 ± 1.3
LDH concn (U/L)				
Median	439.0*	255.0	227.0	68.0
25th–75th percentiles	308.0–645.0	178.5–428.5	131.0–376.0	38.0–103.0
Nucleated cell count (10^9^/L)				
Median	2.7**	0.8	1.4	0.4
25th–75th percentiles	1.3–4.2	0.3–1.4	0.5–3.4	0.2–0.6
Mononuclear cell ratio (%)				
Median	87.6	85.5	90.7	90.0
25th–75th percentiles	77.0–93.7	68.5–92.0	85.0–94.0	75.0–96.9
ADA concn (U/L)				
Median	52.6***	9.2	24.6	6.4
25th–75th percentiles	42.1–63.5	7.6–11.7	10.7–55.4	3.2–21.3
IL-32 concn (ng/L)				
Median	435.4***	130.1	176.7	95.8
25th–75th percentiles	360.3–812.3	79.3–185.1	71.7–244.5	75.2–163.5

a Data are presented as means ± SEM or medians (25th to 75th percentiles). *, *P *< 0.05; **, *P *< 0.01; ***, *P *< 0.001 (compared with each non-TPE group using analysis of variance [ANOVA] followed by Bonferroni’s test). ADA, adenosine deaminase; IL-32, interleukin 32; TPE, tuberculous pleural effusion; MPE, malignant pleural effusion; PPE, parapneumonic pleural effusion.

### Concentration of IL-32 in pleural effusions.

**FIG 1:**
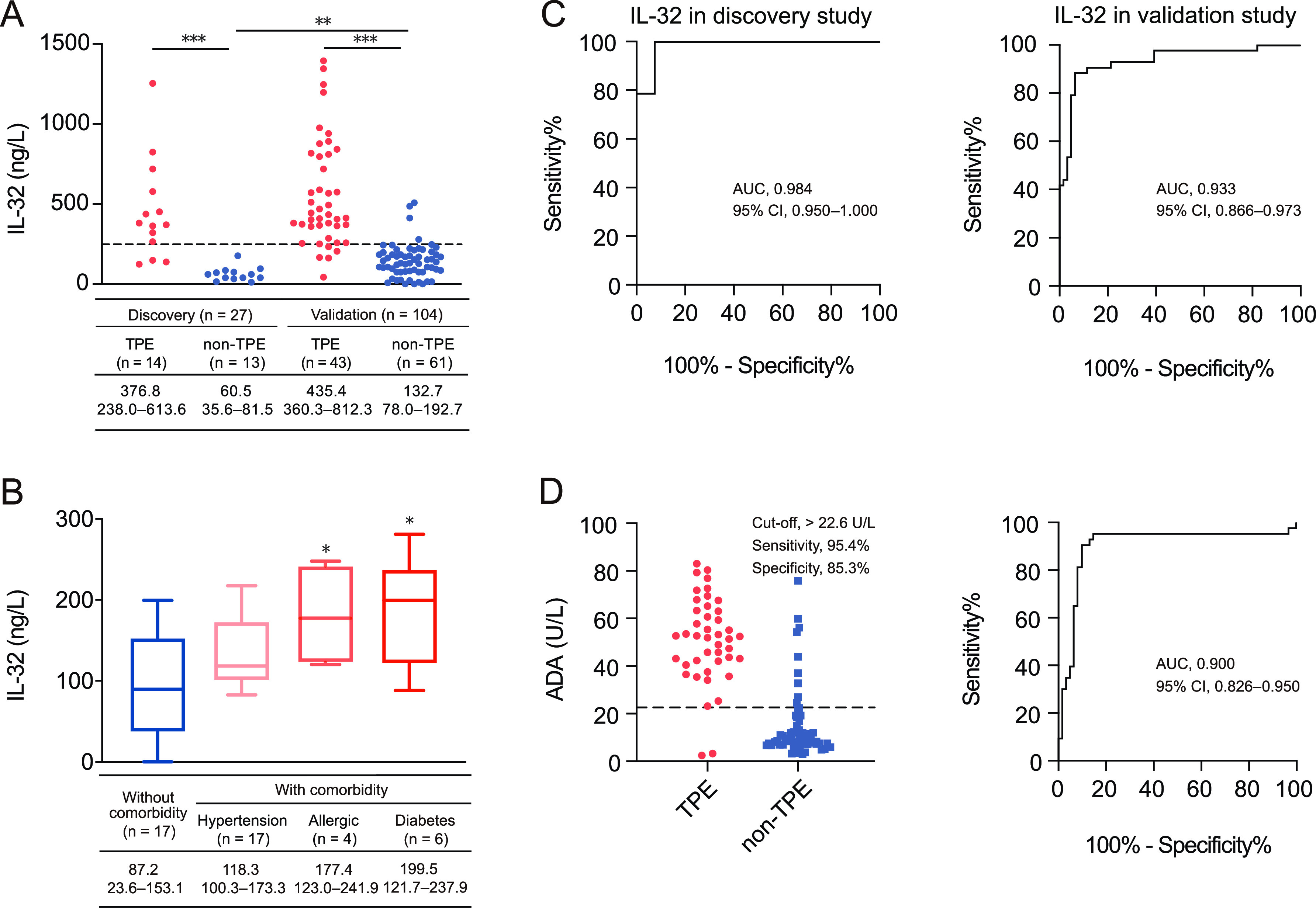
Concentrations and diagnostic accuracies of IL-32 and ADA in pleural effusions. (A) IL-32 levels in pleural effusions in the discovery study and validation study. A cutoff value of 247.9 ng/L for IL-32 showed high sensitivity and specificity for the detection of TPE. (B) IL-32 levels in pleural effusions were significantly upregulated in MPE patients with specific comorbidities. (C) ROC curves showing the diagnostic performance of the IL-32 assay performed on pleural effusion samples in the discovery study and validation study. (D) Scatterplot and ROC curve showing the diagnostic performance of the ADA assay performed on pleural effusion samples. *, *P < *0.05; **, *P < *0.01; ***, *P < *0.001.

In both the discovery study and the validation study, as we hypothesized, the median concentration of IL-32 in TPE was significantly higher than that in non-TPE (Fig. 1), and no difference was observed among malignant, pneumonic, and miscellaneous effusions in the validation study (Table 2). There was no difference in the IL-32 levels in TPE between the two studies, while the IL-32 levels in non-TPE were significantly higher in the validation study than in the discovery study (*P < *0.01) (Fig. 1A). We noted that the concentrations of IL-32 in malignant pleural effusion (MPE) patients with diabetes or allergic disease were significantly higher than those in patients without comorbidities (*P < *0.05), while no significant difference was observed for other comorbidities such as hypertension (*P > *0.05) (Fig. 1B).

### Diagnostic values of IL-32 and ADA in the validation study.

Through the discovery study, we found that IL-32 was a useful diagnostic biomarker for TPE, with an area under the curve (AUC) of 0.984 (Fig. 1C). Next, we further verified this finding in the Beijing cohort and compared its diagnostic potential with that of ADA, a classical marker of TPE. With a cutoff value of 247.9 ng/mL in the receiver operating characteristic (ROC) curve analysis, the AUC of IL-32 to separate TPE from non-TPE was 0.933 (95% confidence interval [CI], 0.866 to 0.973) (Table 3). A similar result was observed for the diagnostic performance of ADA, with a difference between the AUCs of 0.033 (95% CI, −0.047 to 0.112; *z* = 0.807, *P > *0.05) (Fig. 1D).

**TABLE 3:** Diagnostic performances of pleural parameters in differentiating TPE from non-TPE (*n* = 104)^*a*^

Variable	Cutoff value	Median AUC (95% CI)	Median sensitivity (%) (95% CI)	Median specificity (%) (95% CI)	Median PLR (95% CI)	Median NLR (95% CI)	Median PPV (95% CI)	Median NPV (95% CI)
IL-32 (ng/L)	>247.90	0.933 (0.866–0.973)	88.4 (74.9–96.1)	93.4 (84.1–98.2)	13.5 (5.2–35.0)	0.1 (0.1–0.3)	90.5 (77.4–97.3)	91.9 (82.2–97.3)
ADA (U/L)	>22.60	0.900 (0.826–0.950)	95.4 (84.2–99.4)	85.3 (73.8–93.0)	6.5 (3.5–11.9)	0.1 (0.0–0.2)	82.0 (68.6–91.4)	96.3 (87.3–99.5)
Model	>0.31	0.994* (0.954–1.000)	93.0 (80.9–98.5)	98.4 (91.2–100.0)	56.7 (8.1–397.1)	0.1 (0.0–0.2)	97.6 (87.1–99.9)	95.2 (86.7–99.0)

a *, *P *< 0.05 (compared with adenosine deaminase [ADA] using the *z* statistic). The multivariate model included age, ADA, LDH, and interleukin-32 (IL-32). AUC, area under the curve; PLR, positive likelihood ratio; NLR, negative likelihood ratio; PPV, positive predictive value; NPV, negative predictive value.

### A multivariate model with high diagnostic performance.

A multivariate model (named the “TPE screen score”) was constructed to evaluate the diagnostic value of parameter combinations using the binary logistic regression method. IL-32, ADA, lactate dehydrogenase (LDH), and age entered the final multivariate model. The multivariate model is described by the following equation: TPE screen score = (0.030 × IL-32 [ng/L]) + (0.113 × ADA [U/L]) − (0.009 × LDH [U/L]) − (0.282 × age) + 3.579.

**FIG 2:**
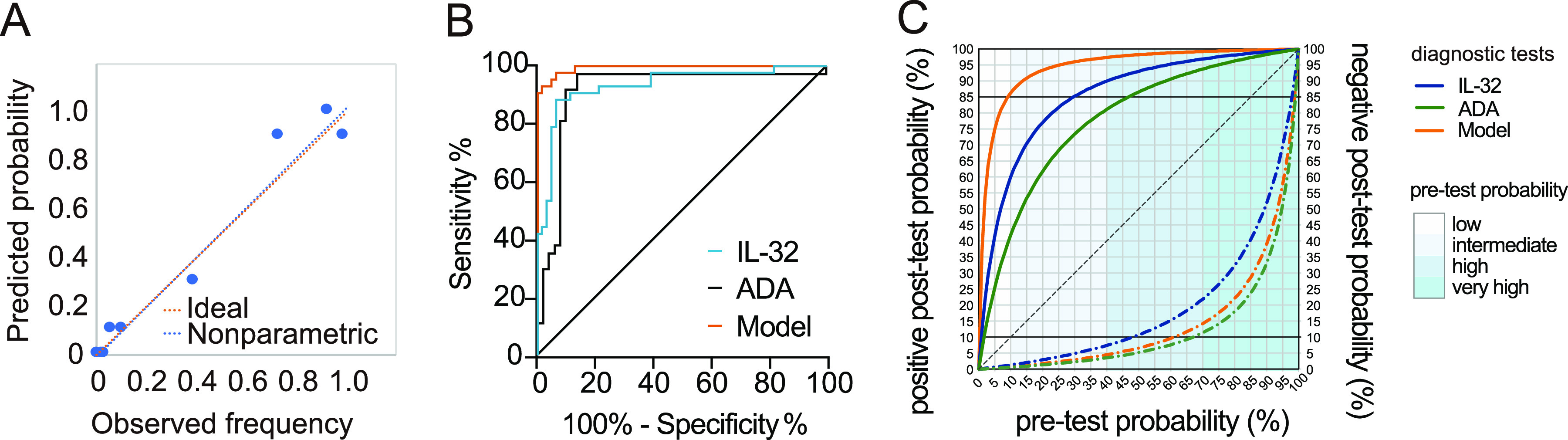
Diagnostic accuracy of the multivariate model and effects of TPE prevalence on the accuracy of diagnostic tests. (A) Calibration plot of the multivariable model. (B) Comparison of the ROC curve of the multivariate model with those of IL-32 and ADA. (C) Bayesian relationship between the pretest probability and posttest probability across diagnostic tests. Line colors represent the three diagnostic tests, and the overlaid blue rectangles represent the pretest probabilities for four groups. Full lines represent a positive posttest probability, and dashed-dotted lines represent a negative posttest probability. The TPE screen score model included age, ADA, LDH, and IL-32.

The test performance of the TPE screen score was accessed, with a score of >0.31 being chosen to classify a positive TPE screen (Table 3). The variance inflation factor (VIF) index of all independent variables was <3, indicating a lack of multiple linear relationships among the predictors. The Hosmer-Lemeshow test *P* value was 0.132, indicating goodness of fit for the model (*P > *0.05). The calibration plot of the observed frequency compared with the predicted probability of the model showed an intercept of −0.013 and a slope of 1.031, suggesting acceptable calibration (Fig. 2A). The comparison of ROC curves showed that the AUC of the model was significantly higher than that of IL-32 or ADA (*P < *0.05), with differences of 0.061 (95% CI, 0.014 to 0.108) and 0.094 (95% CI, 0.021 to 0.167), respectively (Fig. 2B). The scorecard distribution based on the logistic regression model can be found in Table S3. Using the summative score of the model, we can predict the probability of TPE. Scores of >62 were considered to indicate a high risk of TPE, with an AUC of 0.991, a sensitivity of 90.70%, and a specificity of 96.72%.

We analyzed the impact of the estimated pretest probability (prevalence) on the performance of diagnostic tests for TPE by Bayes’s theorem (Fig. 2C). A positive posttest probability of >85% was considered sufficient to rule in TPE. In comparison, a negative posttest probability of <10% was accurate enough to rule out TPE. In low-prevalence settings (prevalence of <10%), the positive posttest probabilities of ADA, IL-32, and the TPE screen score were <85%, which was insufficient to rule in TPE. The TPE screen score presented the highest posttest probabilities in all groups and was sufficient to rule in diagnosis in settings with a pretest probability of over 10%. The negative posttest probability of the model was <10% unless the pretest probability of the disease exceeded 65%. This result shows that the multivariate model is a relatively reliable biomarker to rule in or rule out TPE in intermediate- and high-prevalence settings.

### IL-32 correlates with ADA expression in TPE.

The correlations between IL-32 in pleural effusion and cytological or biochemical parameters of TPE and non-TPE were determined. The level of IL-32 was positively correlated with ADA, LDH, and nucleated cell counts, generally related to total protein in pleural effusions (all *P < *0.001), and negatively correlated with glucose (*P < *0.05) but was not correlated with the mononuclear cell ratio (*P > *0.05) (Fig. S3A). Further analysis showed that IL-32 was positively correlated with ADA and LDH in the TPE group (all *P < *0.01) but not in the non-TPE group (Table S4). Linear regression demonstrated that IL-32 was independently correlated with ADA and LDH in TPE (Fig. S3B).

### IL-32β and IL-32γ positively correlated with ADA2 expression in TPE.

**FIG 3:**
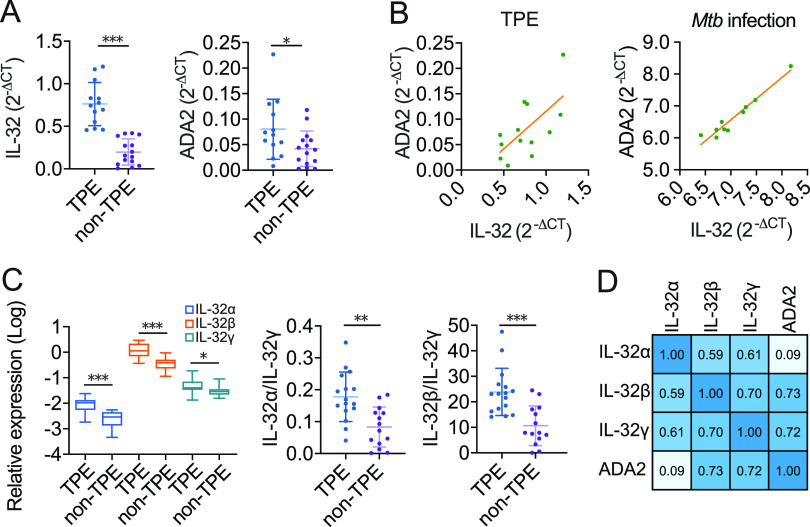
Correlation between IL-32 and ADA2 mRNA expression levels in TPE and non-TPE. RNA was obtained from mononuclear cells in pleural effusions. (A) Total IL-32 and ADA2 mRNA expression levels were significantly enhanced in TPE versus non-TPE mononuclear cells. (B, left) Correlation between and simple linear regression of total IL-32 and ADA2 levels in TPE (*n* = 13) (Pearson *r* = 0.643; *P < *0.05). (Right) The correlation between IL-32 and ADA2 was validated in the transcriptome analysis of GEO data sets (*n* = 10) (Pearson *r* = 0.969; *P < *0.01). *Mtb*, Mycobacterium tuberculosis. (C) The expression of IL-32 isoforms was significantly enhanced in TPE versus non-TPE mononuclear cells. The ratios of IL-32α/IL-32γ and IL-32β/IL-32γ were significantly changed in TPE versus non-TPE mononuclear cells. (D) Spearman correlation matrix of IL-32 isoforms and ADA2 in TPE. Error bars indicate means ± SEM. *, *P < *0.05; **, *P < *0.01; ***, *P < *0.001 (by Student’s *t* test).

TPE has high ADA activity, attributed principally to high levels of the isoenzyme ADA2, which is expressed only by monocytes and macrophages (14, 15). Mononuclear cells in 23 human pleural effusion samples were investigated for total IL-32, IL-32 isoforms (IL-32α, IL-32β, and IL-32γ), and ADA2 mRNA expression, and correlation studies were performed. Total IL-32 was significantly upregulated in the TPE group (all *P < *0.05) (Fig. 3A) and exhibited an excellent diagnostic performance to separate TPE and non-TPE (AUC = 0.978 [95% CI, 0.889 to 0.999]). Total IL-32 mRNA was positively correlated with ADA2 in the TPE group (*n* = 13) (Pearson *r* = 0.643; *P < *0.05) but not in the non-TPE group (*n* = 10) (Pearson *r* = 0.292; *P > *0.05), and this correlation was further verified by the transcriptome expression data (GEO accession number GSE147964) of blood samples from patients infected with *Mtb* (*n* = 10) (Pearson *r* = 0.969; *P < *0.01) (Fig. 3B). Macrophages differentiated from THP-1 cells (Fig. S4A and B) were treated with lipopolysaccharide (LPS), and we found that total IL-32 and ADA2 mRNA expression levels were dependent on the LPS concentration and were positively correlated (*n* = 11) (Pearson *r* = 0.720; *P < *0.01) (Fig. S4C and D).

IL-32α, IL-32β, and IL-32γ mRNA expression levels in mononuclear cells in the TPE group were significantly upregulated, and the alternative splicing of IL-32γ into IL-32α (IL-32α/IL-32γ ratio) and IL-32β (IL-32β/IL-32γ ratio) was significantly enhanced (all *P < *0.05) (Fig. 3C). ADA2 mRNA expression correlated significantly with the mRNA expression of IL-32β and IL-32γ in the TPE group (all *P < *0.05) but not IL-32α (Fig. 3D).

### Roles of endogenous and exogenous IL-32 in regulating ADA2 expression.

**FIG 4:**
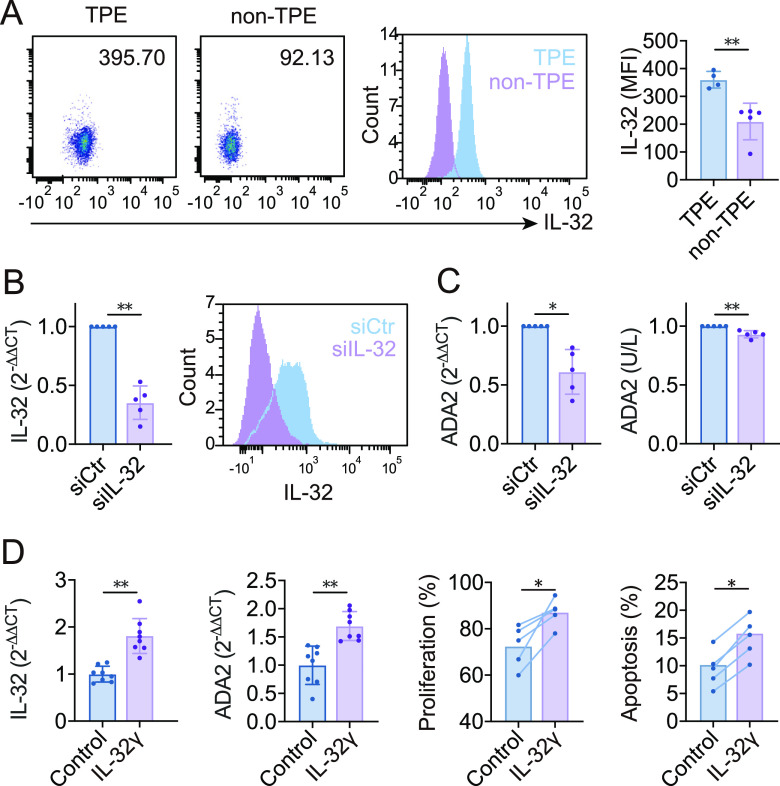
IL-32 staining in macrophages. Knockdown of IL-32 suppressed ADA2 expression and secretion. (A) Density plots and overlay histogram showing that IL-32 expression in macrophages was significantly enhanced in TPE versus non-TPE (*P < *0.01). MFI, mean fluorescence intensity. (B) Macrophages isolated from TPE were transfected with negative-control siRNA (siCtr) or IL-32-specific siRNA (siIL-32), and total IL-32 expression was determined by qRT-PCR and flow cytometry. (C) ADA2 expression levels in IL-32 knockdown macrophages were significantly downregulated. (D) Total IL-32 and ADA2 expression levels were significantly upregulated in macrophages treated with IL-32γ, and Ki67 and an FITC annexin V detection kit were applied to detect proliferation and apoptosis by flow cytometry. Error bars indicate means ± SEM. *, *P < *0.05; **, *P < *0.01 (by Student’s *t* test).

The flow cytometry results revealed that the expression of IL-32 was enhanced in macrophages in TPE compared to non-TPE (*P < *0.01) (Fig. 4A). Macrophages purified from TPE transfected with IL-32 small interfering RNA (siRNA) (Fig. 4B) showed lower levels of ADA2 expression and secretion than did those from the siRNA control group (Fig. 4C). As only the IL-32γ-specific exon contains a potential signal peptide, we hypothesized that IL-32γ is the only isoform that can be secreted (10, 16). IL-32γ-treated macrophages expressed higher IL-32 and ADA2 levels than did untreated cells (Fig. 4D). In addition, the proliferation and apoptosis of IL-32γ-treated macrophages were enhanced.

## DISCUSSION

Tests of pleural effusions are considered minimally invasive methods for differentiating TPE and non-TPE. However, accurate and stable biomarkers in the pleural effusion that can reliably confirm TPE have not been reported thus far, and the diagnostic accuracy of a single biomarker is lower than expected in regions with extreme prevalences (4). In our previous study, we reported that a definitive diagnosis of TPE could be established by medical thoracoscopy in almost all cases (17), but this complex technique may not be accessible in some regions. The purpose of this study was to report a novel biomarker of TPE and evaluate its diagnostic performance.

Our data support IL-32 in pleural effusion as a biomarker with diagnostic value in TPE. The positive likelihood ratio (PLR) value of 13.5 for IL-32, which suggests that patients with IL-32-positive pleural effusion have a 13.5-fold-higher chance of having TPE than non-TPE, is sufficient to include patients. The negative likelihood ratio (NLR) value for IL-32 was 0.1, which is acceptable to exclude TPE. Consistent with data from previous studies, ADA has an excellent sensitivity related to diagnostic performance (5, 15). However, the ADA level is elevated in some inflammatory and lymphoma pleural effusions (18) independent from TPE, leading to a decrease in specificity for differentiating TPE, with >50% of parapneumonic pleural effusion (PPE) cases being misclassified as TPE. To improve diagnostic efficacy, a multivariate diagnostic model comprising IL-32, ADA, LDH, and age was established and was found to have excellent diagnostic performance for discriminating between TPE and non-TPE.

A constant pretest probability is one of the main assumptions for the transferability of test results across settings (19). Previously, we reported that of 833 patients in Beijing with undiagnosed exudative pleural effusion who underwent medical thoracoscopy, 40.0% were eventually diagnosed as having TPE (20). In another study, we recruited consecutive adult patients with pleural effusion in Beijing and Wuhan and found that the TPE prevalences in Beijing and Wuhan were 33.1% and 36.7%, respectively (21). The prevalence of TPE is likely to be lower in developed countries, such as 5.6% in the United Kingdom (22) and 4% in the United States (23), and higher in developing countries such as India (30 to 80%) (24).

As shown in Fig. 2C, when the prevalence of TPE is <10%, the posttest probability of positive results of ADA, IL-32, and the model was <85%, making it unacceptable for “ruling in” TPE. In areas with a >30% prevalence, IL-32 can be used as a biomarker to establish a diagnosis of TPE. In intermediate-prevalence regions (10 to 40%), in a patient with a high clinical suspicion of TPE, a positive model result for an exudative pleural effusion carries a positive posttest probability of >85%. In high- and very-high-prevalence settings, positive model test results increased the probability of TPE to >97.4%, which is considered safe to rule in TPE. This strategic rule makes it possible to reduce the costs and pain to the patient associated with an unnecessary invasive procedure. When the prevalence is <70% and ADA results are negative, the posttest probability is low enough (<10%) to reliably rule out TPE, which prevents the inappropriate administration of antituberculosis treatment to patients with non-TPE.

IL-32γ is the longest and most active isoform of IL-32 and can be spliced into the less active isoforms IL-32β and IL-32α (10, 25). We found that alternative splicing was enhanced in TPE. Previous studies of models of colitis (26) and rheumatoid arthritis (10) have suggested that this alternative splicing of IL-32 plays a role in the self-imposed limitation of uncontrolled inflammation. Therefore, we propose that the increased splicing events generating IL-32β and IL-32α in TPE may contribute to the self-limitation of inflammation in TPE and may be related to prognosis.

IL-32 and ADA2 have similar biological roles in promoting tumor necrosis factor alpha (TNF-α) production and inducing macrophage differentiation (27, 28); thus, we hypothesized that IL-32 and ADA2 are involved in the same signaling pathway in TPE. Furthermore, the correlation between IL-32 and ADA2 expression levels in TPE was verified at both the protein and mRNA levels. Based on experiments involving transfecting IL-32-specific siRNA into macrophages and culturing macrophages with IL-32γ, we speculated that both endogenous IL-32 and exogenous IL-32γ could regulate the expression of ADA2.

Effector T cells are critically important for antituberculosis immunity (29), but the role of IL-32 in T cell-mediated immunity in TPE is unclear. An association between IL-32 and T cells has been reported in several studies. For instance, T cells in lung tissue in IL-32γ-transgenic mice infected with *Mtb* expressed higher levels of interferon gamma (IFN-γ) and TNF-α (11), and IL-32 was correlated with the balance of T_H_1/T_H_17 cytokines in the peripheral blood of tuberculosis patients (30). Although there is no evidence that the IL-32 receptor proteinase 3 is expressed on the surface of T cells (31), several studies have shown that IL-32 modulates T cell immune responses through macrophages and dendritic cells (DCs), such as IL-32 activating intratumoral DCs and macrophages that recruit CD8^+^ T cells in melanoma (32) and IL-32 inhibiting CD4^+^ T cell proliferation in multiple myeloma through macrophages (33). Our study suggests that IL-32 promotes macrophage secretion of ADA2, which was reported to bind directly to the surface of T cells and stimulate T cell proliferation (28, 34).

It should be noted that this study had several limitations. First, not all patients diagnosed with TPE were assessed based on bacteriological or histological results but rather were assessed based on clinical characteristics and the effectiveness of antituberculosis therapy, which might have led to misclassification and prevented the results from reflecting the true accuracy of the test. In addition, diagnostic evaluations were performed on a limited number of patients, resulting in wide confidence intervals for the estimated sensitivity or specificity of the results, which limited the generalizability of the test results. Therefore, the diagnostic accuracy of IL-32 in pleural effusion and the multivariate model for TPE diagnosis needs to be further verified in subsequent concurrent multicenter cross-sectional studies.

### Conclusions.

We demonstrate that a high IL-32 level in the pleural effusion is a valuable biomarker for identifying patients with TPE. A multivariate model combining age, ADA, LDH, and IL-32 may reliably rule in TPE in intermediate- or high-prevalence areas. Furthermore, our results suggest that IL-32β is the main isoform in mononuclear cells and that the splicing of IL-32γ into IL-32α and IL-32β is enhanced in TPE, which may indicate a negative feedback loop for inflammation (10). The correlation between IL-32 and ADA expression revealed the regulation of ADA expression in macrophages by IL-32 in TPE, which was further confirmed by *in vitro* experiments involving human macrophages and the THP-1 cell line. The mechanism by which IL-32 induces ADA expression remains unclear and requires further research. Thus, the current study provides novel insight into the role of IL-32 in the tuberculosis microenvironment.

## MATERIALS AND METHODS

### Participants.

From January 2018 to December 2019, patients with undetermined pleural effusion were consecutively recruited from Wuhan Union Hospital and Beijing Chao-Yang Hospital in this prospective study. The Wuhan cohort (discovery study) was designed to detect the IL-32 levels in pleural effusions to provide a baseline (35). Patients without known comorbidities were included in this cohort. The Beijing cohort (validation study) was used to validate the diagnostic efficacy of IL-32 for TPE and assess the impact of comorbidities on IL-32 levels. All of the included patients met the inclusion criteria listed below, while those meeting the exclusion criteria were excluded. Before the collection of pleural effusion samples, each patient was informed and provided written informed consent. This study has been approved by the ethics committees of Wuhan Union Hospital (2019-S880) and Beijing Chao-Yang Hospital (2018-KE-321). The diagnostic accuracy study section was designed according to STARD (Standards for Reporting of Diagnostic Accuracy) guidelines (36, 37).

### Diagnostic criteria.

TPE was diagnosed if one of the following criteria was met: demonstration of caseating granulomas and/or epithelioid cell granulomas in pleural tissue, with no evidence of other granulomatous diseases; positive acid-fast staining; a positive *Mtb* culture from pleural effusion, pleural biopsy, or sputum specimen; or the presence of clinical data highly suggestive of tuberculosis, with an improvement in the pleural effusion after antituberculosis therapy. Malignant pleural effusion (MPE) was considered if malignant cells were observed in the pleural effusion or pleural biopsy specimens. Parapneumonic pleural effusion (PPE) was classified as pleural effusion associated with bacterial pneumonia, lung abscess, or bronchiectasis. Miscellaneous pleural effusion was described as pleural effusion caused by other diseases. The exclusion criteria were antituberculosis or antitumor therapy before admission, an immunosuppressed status (HIV infection, hepatitis B/C virus infection, or hormonal or immunosuppressive therapy), invasive intrathoracic procedures or chest trauma within 3 months, an undetermined diagnosis, or empyema and transudate effusion.

### Measurement of IL-32 and ADA.

Pleural effusion was collected via diagnostic thoracentesis or medical thoracoscopy before patients received any treatment. The concentration of IL-32 in the pleural effusion was determined using the sandwich enzyme-linked immunosorbent assay (ELISA) method (R&D Systems, USA) according to the manufacturer’s protocols. ADA activity in the pleural effusion was determined using a Beckman automatic biochemical apparatus (Beckman Coulter, USA), using colorimetric method kits (In Tec Products, China). Each sample was used only once to prevent protein degradation due to repeated freeze-thaw processes.

### Flow cytometry.

The flow cytometry antibodies CD14-eFluor 450 and CD68-fluorescein isothiocyanate (FITC) were purchased from eBioscience, Ki67-allophycocyanin (APC) was purchased from BioLegend, and IL-32–Alexa Fluor 488 was purchased from R&D Systems. An FITC annexin V apoptosis detection kit purchased from BD was used to detect the cell apoptosis fraction. Mononuclear cells isolated from TPE or macrophages differentiated from THP-1 cells were stained for flow cytometry as previously described (38) to analyze the expression of surface markers, intracellular markers, apoptosis, and proliferation incorporation using the three-laser FACSAria II system (BD Biosciences, USA). Marker staining was performed according to the manufacturer’s protocol.

### RNA isolation and quantitative real-time PCR.

Total RNA was extracted using TRIzol (Tiangen, China). Genomic DNA removal and cDNA synthesis were performed using a reverse transcription kit (TaKaRa Bio, China). SYBR green I master mix (Roche, Switzerland) was used for quantitative real-time PCR (qRT-PCR). The glyceraldehyde-3-phosphate dehydrogenase (GAPDH) gene was selected as an internal control gene. Each sample was assayed in duplicate. The primer sequences used are listed in Table S5 in the supplemental material.

### Cell isolation and transfection assay.

We purified CD14^+^ cells from TPE using a human CD14^+^ cell isolation kit (Miltenyi Biotec, Germany) and cultured the cells (2 × 10^6^ cells/mL) in complete RPMI 1640 medium containing 10% fetal bovine serum (FBS). CD14^+^ cells were transfected with small interfering RNA (siRNA) or a negative control (100 nM; RiboBio, China) using Lipofectamine RNAiMAX (Life Technologies, USA).

### Cell culture and differentiation.

THP-1 cells (ATCC TIB-202) were inoculated into 48-well plates and cultured for 24 h in the presence of 15 ng/mL phorbol 12-myristate 13-acetate (PMA) at 37°C in a humidified atmosphere with 5% CO_2_ for adhesion and differentiation. After incubation overnight, the medium containing PMA was replaced with fresh PMA-free RPMI 1640 plus 10% FBS and 2 mM glutamine.

### Statistical analysis.

The sample size was calculated using PASS 15 (Power Analysis and Sample Size Software, USA). According to the Shapiro-Wilk test, normally distributed variables are presented as the means ± standard errors of the means (SEM), and nonnormally distributed variables are presented as the medians and 25th to 75th percentiles. Significant differences in categorical variables were determined using the chi-square test. In addition, Student’s *t* test, the Mann-Whitney U test, one-way analysis of variance (ANOVA), or Kruskal-Wallis ANOVA was performed to test differences between two or multiple groups, as appropriate. Pearson and Spearman correlation coefficients were computed for the relationship between IL-32 and all variables. A simple linear regression was performed to check the parameters correlated with IL-32. Receiver operating characteristic (ROC) curves were drawn, and the areas under the curve (AUCs) were calculated and compared using *z* statistical analysis. Finally, a multivariate model was constructed using the binary logistic regression method. All statistical analyses were performed using GraphPad Prism 8.0 (GraphPad Software, USA) and MedCalc 15.2 (MedCalc Software, Belgium). A *P* value of <0.05 was considered statistically significant.

### Data availability.

Relevant files of this work will be shared upon reasonable request.
