# Supplementary material for: Interleukin 32 as a Potential Marker for Diagnosis of Tuberculous Pleural Effusion
Source: Microbiol Spectr. 2022 Jul 26;10(4):e02553-21. doi: 10.1128/spectrum.02553-21 (PMC9430160; doi:10.1128/spectrum.02553-21)
Supplement: Supplemental file 1 — Supplemental material. Download spectrum.02553-21-s0001.pdf, PDF file, 0.5 MB [file spectrum.02553-21-s0001.pdf]

Supplementary Tables

Supplementary Table 1. Cause distribution of pleural effusions in this study

Variables	Discovery study (n = 27)	Validation study (n = 104)
Tuberculous pleural effusion	14	43
Malignant pleural effusion	10	41
Lung cancer	6	33
Mesothelioma	1	4
Breast cancer	0	2
Esophagus cancer	1	0
others	1	0
unknown	1	2
Infectious pleural effusion	2	13
Miscellaneous	1	7
Systemic lupus erythematosus	0	1
Pulmonary embolism	0	1
COPD	0	2
others	1	3

Supplementary Table 2. Pathological findings of pleural biopsy specimen of patients with tuberculous pleural effusion

Variables	Discovery study (n = 14)	Validation study (n = 43)
Caseating granulomas		
Caseating granulomas	1	3
Caseating granulomas + Acid-fast bacillus	1	2
Epithelioid cell granuloma		
Epithelioid cell granuloma	4	14
Epithelioid cell granuloma + Acid-fast bacillus	2	6
Caseating granulomas + Epithelioid cell granuloma		
Caseating granulomas + Epithelioid cell granuloma	2	6
Caseating granulomas + Epithelioid cell granuloma + Acid-fast bacillus	4	9
Empirical treatment	0	3

Supplementary Table 3. Points assigned to patient-level variables

Variables		Points
Age	≤ 44	21
	> 44	-20
ADA	> 22.56	17
	≤ 22.56	-27
LDH	≤ 285	42
	285−1500	28
	> 1500	-50
IL-32	≤ 247.92	-24
	> 247.92	31

Base score is 20 and the risk increases/decreases based on the variables in the table.

Supplementary Table 4. Correlation and association of IL-32 with continuous variables

Variables	TPE		non-TPE	
	Spearman r	B (95% CI)	Spearman r	B (95% CI)
ADA	0.447**	0.017* (0.001−0.035)	0.148	
LDH	0.500**	0.302* (0.070−0.533)	0.088	
Protein	-0.237		0.001	
Glucose	-0.237		-0.211	
Nucleated cell counts	0.377*	0.002 (0.000−0.005)	0.067	

Abbreviations: ADA, adenosine deaminase; LDH: Lactate dehydrogenase; TPE, tuberculous pleural effusion.

*P < 0.05, **P < 0.01.

Supplementary Table 5. PCR primer sequences used in this study

Gene		Primer Sequence (5' − 3')
GAPDH	Forward	GGAGCGAGATCCCTCCAAAAT
	Reverse	GGCTGTTGTCATACTTCTCATGG
β-actin	Forward	CATGTACGTTGCTATCCAGGC
	Reverse	CTCCTTAATGTCACGCACGAT
IL-32 total	Forward	AGGACGTGGACAGGTGATGTC
	Reverse	GTCTCCAGGTAGCCCTCTTTGA
IL-32α	Forward	CACCCAGAGCTCACTCCTCT
	Reverse	GGCTCCGTAGGACTTGTCAC
IL-32β	Forward	GAAGACTGCGTGCAGAAGGT
	Reverse	CTTTCTATGGCCTGGTGCAT
IL-32γ	Forward	GGTAATGCTCCTCCCTAC
	Reverse	GAGGAGTGAGCTCTGGGTGC
ADA2	Forward	ACCATGACGAAGAGTGGTCAG
	Reverse	CCATTCGGATGGATTCTGCG

Supplementary Figures

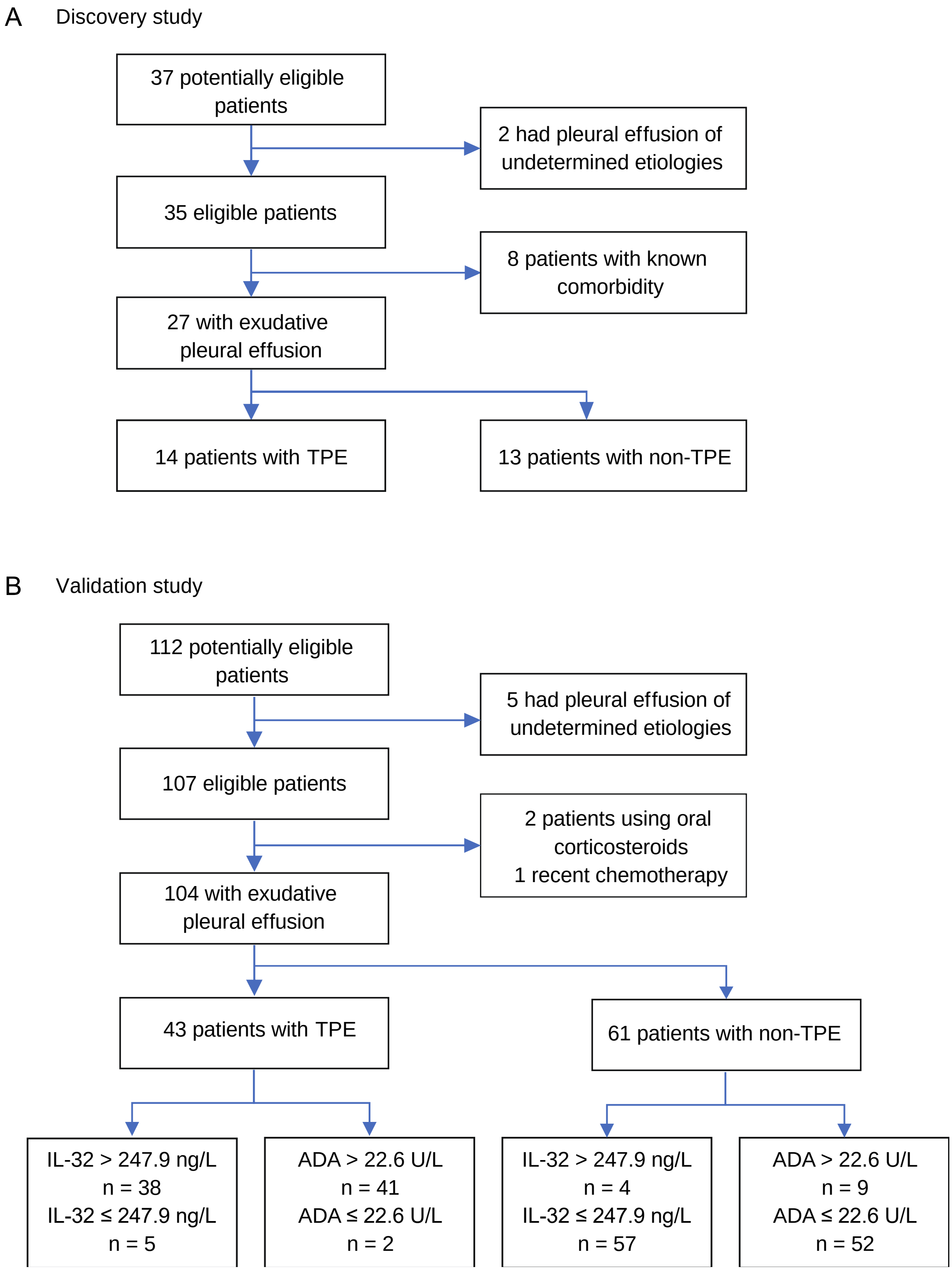

Supplementary Figure 1. Flow diagram of the study population in discovery study (A) and validation study (B). IL-32: interleukin 32; ADA, adenosine deaminase.

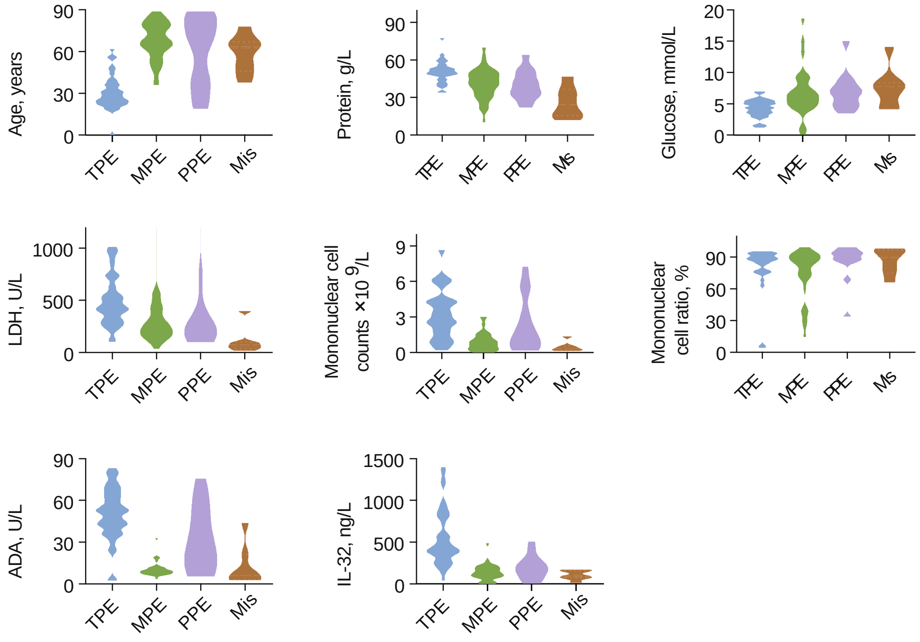

Supplementary Figure 2. Violin plots show the distributions of characteristics of pleural effusions in validation study.

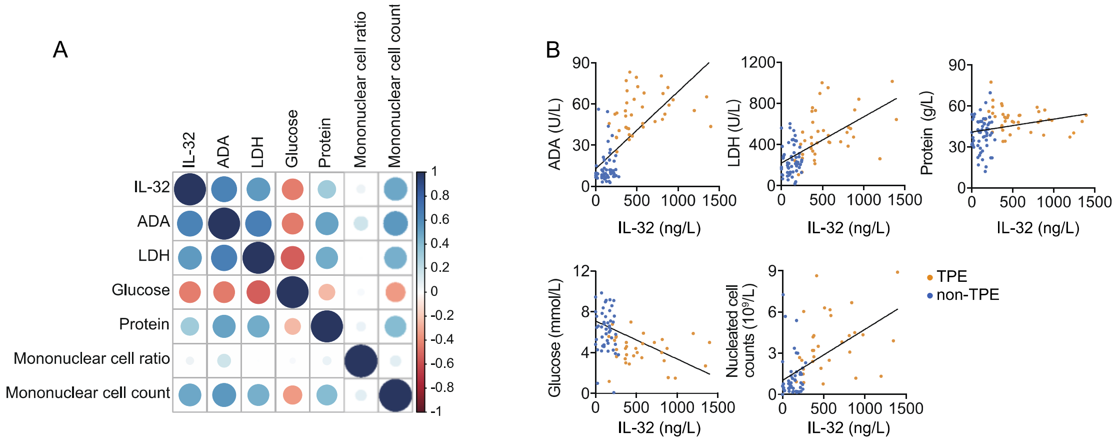

Supplementary Figure 3. Correlation of IL-32 protein level with cytological parameters and biochemical parameters in pleural effusion samples. (A) Spearman correlation of IL-32 with continuous variables in all pleural effusion samples. (B) Simple linear regression of IL-32 with ADA, LDH, protein, glucose, nucleated cell counts, and mononuclear cell ratio in all pleural effusion samples.

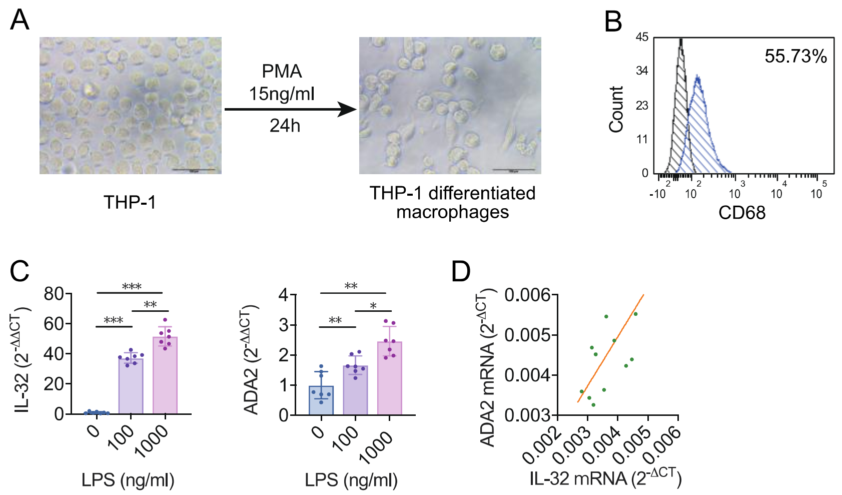

Supplement Figure 4. THP-1 differentiated macrophages after stimulated by LPS for 24 hours. (A) Macrophages differentiated from THP-1 by PMA for 24 hours had a morphology change. Optical microscopy, 400×. (B) The levels of macrophage marker CD68 were determined by flow cytometry. (C) Total IL-32 and ADA2 mRNA expression levels of THP-1 differentiated macrophages. (D) Correlation and simple linear regression of total IL-32 and ADA2 expression (n = 11, Pearson r = 0.720, P < 0.01). Data are presented as mean ± SEM. *P < 0.05, **P < 0.01, ***P < 0.0001.
